# Agronomic Performance and Stability of Vegetable Amaranth (*Amaranthus spp*.) Genotypes in Benin

**DOI:** 10.1002/pei3.70076

**Published:** 2025-08-07

**Authors:** Mathieu A. T. Ayenan, Lys A. Aglinglo, Lydi C. Amoussou, Roland M. Sossa, Chedrac Zokpon, Fekadu Fufa Dinssa, Roland Schafleitner

**Affiliations:** ^1^ World Vegetable Center West and Central Africa, Coastal and Humid Regions Cotonou Benin; ^2^ Université Nationale D'agriculture Ketou Republic of Benin; ^3^ World Vegetable Center Eastern and Southern Africa Arusha Tanzania; ^4^ World Vegetable Center Mexico Office Hosted by CIMMYT Texcoco México

**Keywords:** AMMI, GGE, multi‐trait selection index, variety release

## Abstract

Amaranth is a vegetable and grain crop with the potential to contribute to nutrition security and income generation. However, amaranth production in Benin faces several constraints, including the limited availability of improved varieties and access to high‐quality seed. The study aimed to (i) identify top‐performing varieties based on agronomic traits and (ii) identify farmer‐preferred amaranth traits and genotypes to guide variety recommendations for release. Ten amaranth genotypes were evaluated in five environments defined as the combination of locations (Abomey‐Calavi and Malanville) and years (2021, 2022, and 2023). Traits prioritization and variety ranking were done involving 41 experienced amaranth farmers. The analysis of variance revealed a significant genotype‐by‐environment interaction for fresh biomass yield, leaf length, and leaf width. Genotype was a major determinant of leaf length and leaf width, but had a lesser effect on fresh biomass yield. Genotypes A2002, IP‐5‐Sel, Madiira1, and Nguruma exhibited the most stable yield across environments. IP‐5‐Sel, A2004, and AC‐NL were the most stable genotypes for leaf width, while IP‐5‐Sel and Madiira1 were the most stable for leaf length. The five environments formed a single mega‐environment for fresh biomass yield and leaf width. The multi‐trait selection index identified Nguruma and Madiira2 as the top performers when considering fresh biomass yield and leaf parameters. Farmer‐preferred traits included marketability, branching, late flowering, and cooking quality. Based on these traits, the most preferred genotypes were Madiira2, AC‐NL, Nguruma, and Akeri. Combining the agronomic and farmer preferences, and the release status of the genotypes in West Africa, we recommended Madiira2, IP‐5‐Sel, Nguruma, AVAM1939, AC‐NL, and Akeri for release. The lines have been channeled into the variety release process in Benin.

## 1 Introduction

Amaranth (*Amaranthus spp*) is a traditional vegetable widely grown and consumed for its fresh leaves, young shoots, and grains. Amaranth is one of the most important cultivated and consumed leafy vegetables in Benin (Dansi et al. 2008, Sogbohossou et al. 2015). Amaranth leaves are rich in beta‐carotene, iron, calcium, protein, and dietary fiber (Sarker et al. 2020; Venskutonis and Kraujalis 2013). Owing to their nutritional composition, amaranth leaves have beneficial health effects (Baraniak and Kania‐Dobrowolska 2022; Sarker et al. 2022). The crop is fast‐growing, with the first harvest possible 30 days after sowing, requiring minimal investment from farmers, and providing quick returns on investment. Amaranth has important cultural acceptance across many African countries (Sogbohossou et al. 2015). Amaranth is integrated into crop rotation systems for its ability to reduce nematode populations (Grubben 2004; Sogbohossou et al. 2015).

Despite its potential nutritional and economic importance, amaranth production in Africa is constrained by little investment in research, the low availability of improved varieties, and the predominance of farmers' seed systems. The crop is perceived in some communities as a “poor man vegetable” while in some regions amaranth consumption is very popular, and demand is rising (Nyonje et al. 2022; Dinssa et al. 2022). Access to planting materials is one of the barriers to promoting amaranth value chains (Karl et al. 2024). For instance, as of October 1, 2024, there was no record of released amaranth varieties in Benin (MAEP 2016), Senegal (MAER 2012), Ghana (MoFA (Ministry of Food and Agriculture) 2019), as well as in the Economic Community of West African States (ECOWAS) regional variety Catalogue (CEDEAO‐CILSS‐UEMOA 2022). This situation limits farmers' choices of varieties available in the formal seed sector, making it challenging for farmers to meet diverse market requirements. Local seed production and commercialization require prior variety release and registration in national or regional catalogs. Considering the growing importance of amaranth in Benin, and to sustain the development of amaranth value chains, it is crucial to provide farmers with a diverse portfolio of varieties meeting the market needs and adapted to local growing conditions. Improved amaranth genotypes have been developed at the World Vegetable Center (Dinssa et al. 2022), and their introduction in West Africa can help farmers access high‐performing genotypes while increasing the portfolio of available varieties. Successful variety introduction requires adaptability tests to evaluate their performance under local growing conditions and assess whether the varieties match the prioritized stakeholder traits. To this end, ten amaranth genotypes were introduced in Benin in 2021 and evaluated over years and locations.

Agro‐ecological conditions and management practices affect variety performance. Plant breeders are interested in genotype‐by‐environment interaction as they try to manage, exploit, or ignore it (Annicchiarico 2002). Identifying stable varieties across environments or those adapted to a specific environment is crucial for recommending new varieties for release. Significant changes in genotype performance across environments indicate genotype‐by‐environment (G × E) interaction (Bänziger and Cooper 2001). Change in rank of genotypes across environments has received special attention as it precludes selection based solely on mean performance (Kang 1988) and prompts assessment of the stability of the materials under evaluation. Previous studies on amaranth indicated the influence of environments on genotype adaptability (Dinssa et al. 2022; Dinssa et al. 2019).

Statistical methods have been developed to aid in analyzing the interaction between G and E. Two of these methods, Additive Main effects and Multiplicative Interaction (AMMI) (Gauch and Zobel 1988) and GGE biplot (Yan et al. 2001) have been widely used. AMMI integrates principal component analysis and analysis of variance. As such, it first describes the additive main effects of environments and genotypes by analysis of variance. Secondly, it assesses the interaction between environment and genotypes, which is the non‐additive part, by principal component analysis (Gauch 2013). AMMI is useful in informing on whether there is a significant main effect, a significant G × E interaction of the main effects, delineating mega‐environment, and identifying winning genotypes in each mega‐environment (Gauch 2013). GGE biplot is popular for its ability to pinpoint the best performing genotypes in an environment, to identify stable genotypes in a mega environment, inform about the discriminating (informative) ability of the environment, and the representativeness of testing environments (Yan et al. 2001). Besides these two methods, Olivoto, Lúcio, daSilva, Marchioro, et al. (2019) proposed the best linear unbiased predictor (BLUP)‐based method to assess and visualize G × E interaction.

Variety performance is not only defined by a single trait (yield), especially in leafy vegetables, for which leaf size, shape, and color are essential traits. We hypothesized that the new amaranth genotypes exhibit significant genotype‐by‐environment (G × E) interactions in Benin, necessitating adaptability assessments to identify high‐performing and stakeholder‐preferred varieties suitable for local conditions. The present study used a combination of AMMI, GGE, and BLUP‐based methods, aiming to i) identify top‐performing varieties based on agronomic traits and ii) identify farmer‐preferred amaranth traits and genotypes to guide variety recommendations for release.

## 2 Materials and Methods

### 2.1 Genotypes

The amaranth genotypes used in the current study were obtained from the World Vegetable Center (WorldVeg) amaranth breeding program and included eight *Amaranthus cruentus* and one genotype each of *A. hypochondriacus* and *A. dubius.* All except one genotype have already been released in one or more countries other than Benin (Table 1). In this study, we did not intend to compare the performance of the species but rather the performance of the genotypes regardless of the species, for possible release in Benin. Besides, only the elite materials from the breeding program were included in this study. There were more *A. cruentus* genotypes because this species is more adapted to biomass production and has received more breeding attention in this regard.

**TABLE 1:** Type and origin of 10 amaranth genotypes evaluated across locations and years in Benin.

Genotype	Species	Type	If released (where)^a^	Origin
Madiira 1	*A. cruentus*	Variety	Tanzania, Ethiopia, Kenya	WorldVeg, Tanzania
Madiira 2	*A. cruentus*	Variety	Tanzania	WorldVeg, Tanzania
Akeri	*A. cruentus*	Variety	Tanzania	WorldVeg, Tanzania
Nguruma	*A. dubius*	Variety	Tanzania	WorldVeg, Tanzania
Poli	*A. hypochondriacus*	Variety	Tanzania	WorldVeg, Tanzania
IP‐5‐Sel	*A. cruentus*	Line	—	WorldVeg, Tanzania
AVAM1939	*A. cruentus*	Variety	Tanzania	WorldVeg, Tanzania
AC‐NL	*A. cruentus*	Variety	Cameroon, Ethiopia	WorldVeg, Tanzania
A2002	*A. cruentus*	Variety	Mali	WorldVeg, Mali
A2004	*A. cruentus*	Variety	Mali	WorldVeg, Tanzania

^a^ Record based on partners' feedback to the World Vegetable Center Amaranth breeding program.

### 2.2 Testing Environment, Experimental Design, and Crop Management

The study was conducted in two locations in Benin, namely Abomey‐Calavi and Malanville, over three years, 2021, 2022, and 2023. Considering the combination of each location and year as an independent environment, the genotypes were evaluated in five environments. The geographical coordinates, start (transplanting), and end date (last harvest) of the trials were used as input to retrieve the daily weather data for six variables from NASA POWER (Table 1). We used the combination of location (Abomey‐Calavi and Malanville) and year to define five trial environments (Table 2). Abomey‐Calavi and Malanville fall within two contrasting agroclimatic regions: the Guinean (Abomey‐Calavi) and the Sudanian (Malanville) (Adomou 2005), which are the major areas for vegetable production in the country.

**TABLE 2:** Characteristics of the trial environments, two trial locations, and 3 years in Benin.^a^

Location characteristics	Abomey‐Calavi 2021 (ABC21)	Abomey‐Calavi 2022 (ABC22)	Malanville 2022 (Malan22)	Abomey‐Calavi 2023 (ABC23)	Malanville 2023 (Malan23)
Sowing date	08 Aug 2021	16 Jun 2022	28 Jun 2022	23 Mar 2023	29 Nov 2023
Transplanting date	2 Sep 2021	8 Jul 2022	04 Aug 2022	6 Apr 2023	22 Dec 2023
Trial end (Last harvest)	10 Dec 2021	16 Oct 2022	01 Nov 2022	15 Jun 2023	03 Mar 2024
PAR Total (W/m^2^)	91.56 ± 20.27	82.73 ± 19.31	101.44 ± 21.76	91.83 ± 23.70	107.24 ± 9.24
Temperature at 2 m (°C)	26.50 ± 0.66	25.08 ± 0.52	25.93 ± 1.11	27.40 ± 0.52	27.03 ± 3.47
Relative humidity at 2 m (%)	87.15 ± 2.35	88.16 ± 1.90	81.44 ± 6.76	86.16 ± 2.35	18.97 ± 6.48
Cumulated precipitation corrected during the trial (mm)	521	541	529.95	429	0.07
Temperature at 2 m maximum (°C)	28.66 ± 0.95	27.43 ± 0.76	30.57 ± 1.82	29.31 ± 0.79	37.07 ± 3.18
Temperature at 2 m minimum (°C)	24.86 ± 0.65	23.45 ± 0.64	22.01 ± 1.81	25.90 ± 0.55	18.51 ± 3.45

^a^ Abomey‐Calavi (lat. 6.4195°N, long. 2.3298°E, altitude 20 m, ferralitic soil), Malanville (lat. 11.7707°N, long. 3.5084°E, altitude 192 m, sandy soil); Daily: The total Photosynthetically Active Radiation (PAR) incident on a horizontal plane at the surface of the earth under all sky conditions.

The experiment was laid out in a randomized complete block design with three replications in each environment.

The seedlings, raised on wooden seedling trays in Abomey‐Calavi and on a nursery bed in Malanville, were transplanted at the spacing of 20 cm between rows and plants within rows, i.e., a density of 250,000 plants per hectare. One seedling was transplanted per hole, and there were, on average, 180 plants per experimental unit. Because the experiments were conducted on ferralitic and sandy soils characterized by low organic matter, before transplanting, poultry manure was applied at a rate of 10 t/ha, and additional manure application was made at the same rate 1 week after each harvest. Urea (46% nitrogen) and NPK (15‐15‐15) were applied at 100 kg/ha and 150 kg/ha, two and four weeks after transplanting, following the national guidelines for amaranth production (Mensah et al. 2019). Plants were irrigated, and plots were hand‐weeded when needed.

### 2.3 Data Collection

Time to flowering was recorded as extra‐early (days to first flower ≤ 20 days after transplanting), early (days to first flowering > 20 days and ≤ 40 days), medium early (days to first flowering > 40 days to ≤ 60 days after sowing), and late flowering (genotypes that took > 60 days after sowing). Twenty plants per plot were kept to record the flowering time. We also recorded the flowering time of the regenerated plants after the first cutting.

Leaf length (blade + petiole) was recorded on four randomly selected leaves from four plants per plot at each harvest, and the data were averaged per plot. Similarly, leaf width was measured in the middle of each four randomly selected leaves from four plants at each harvest, and the data were averaged per plot.

Fresh biomass yield per plot was determined by harvesting (cutting) tender stems per plot at 10 cm above ground. The number of harvests during the crop‐growing season ranged from one to four, depending on the environment and genotype. The fresh biomass weight (= yield) was recorded for each harvest. The fresh biomass weight was accumulated across harvests to compute the total biomass per plot. To account for the variability in the number of harvests, the average fresh biomass (leaves and tender stems) weight per harvest was computed by dividing the total weight per plot by the number of harvests per genotype and used to estimate the yield (t/ha) per harvest.

### 2.4 Data Analysis

#### 2.4.1 Within‐Environment Analysis of Variance

Leaf length, leaf width, and fresh biomass yield were subjected to environment‐specific (individual) analysis of variance using Equation (1). We checked the assumptions of the analysis of variance. Genotypes and environments were considered fixed factors.

(1) $$y_{\mathrm{ij}}=\mu +\mathrm{Gi}+\text{Repj}+\varepsilon_{\mathrm{ij}}$$

where $y_{\mathrm{ij}}$ is the *i*th observation in the *j*th replication, *μ* is the overall mean, G is the *i*th genotype, Rep is the *j*th replication, and $\varepsilon_{\mathrm{ij}}$ is the residual.

#### 2.4.2 Combined Analysis of Variance

Leaf length, leaf width, and fresh biomass yield were subjected to a combined analysis of variance to assess whether there was significant variation among genotypes, environments, and the interaction of genotype and environment. Genotypes and environments were considered fixed factors for the combined analysis of variance following Equation (2).

(2) $$y_{\mathrm{ij}}=\mu +\mathrm{Gi}+\mathrm{Ej}+\mathrm{Rep}\left(E\right)\mathrm{jk}+\text{GxEij}+\varepsilon_{\mathrm{ij}}$$

Where $y_{\mathrm{ij}}$ is the *i*th observation in the *j*th environment, *μ* is the overall mean, G is the *i*th genotype, *E* is the *j*th the environment, $\mathrm{Rep}\left(E\right)$ is the *k*th replication within the *j*th environment, G × E is the interaction between the *i*th genotype and the *j*th environment and $\varepsilon_{\mathrm{ij}}$ is the residual effect.

#### 2.4.3 Stability Analysis

To better assess the interactions between genotypes and environments, stability of yield and leaf morphology, and relationships among environments, we used the additive main effects and multiplicative interaction (AMMI) (Gauch 2013) model. We used Equation (3) for the AMMI analysis:

(3) $$y_{\mathrm{ij}}=\mu +\alpha_{i}+\tau_{j}\;\sum_{k=1}^{p}\lambda_{k}a_{\mathrm{ik}}t_{\mathrm{jk}}+\rho_{\mathrm{ij}}+\varepsilon_{\mathrm{ij}}$$

where $\lambda_{k}$ is the singular value for the *k*th interaction principal component axis (IPCA); $a_{\mathrm{ik}}$ is the *i*th element of the *k*th eigenvector; $t_{\mathrm{jk}}$ is the jth element of the *k*th eigenvector. A residual ρ*_ij_* remains, if not all p IPCA are used, where *ρ* ≤ −g‐1; e‐1. AMMISOFT software (Gauch and Moran 2017) was used for the AMMI analysis, model diagnostic, and selection.

To further investigate the structure of genotype‐by‐environment interactions, we computed the weighted Average of Absolute Scores from the best linear unbiased prediction (BLUP) interaction matrix (WAASB) (Olivoto, Lúcio, daSilva, Sari, and Diel 2019). WAASB quantifies the stability of *g* genotypes evaluated in *e* environments using a linear mixed‐effect model. WAASB is computed considering all Interaction Principal Component Axes (IPCA) from the Singular Value Decomposition (SVD) of the matrix of genotype‐environment interaction (GEI) effects generated by a linear mixed‐effect model, as follows:

$${\text{WAASB}}_{i}=\sum_{k=1}^{p}|{\text{IPCA}}_{\mathrm{ik}}X\;\mathrm{EPk}|/\sum_{k=1}^{p}{\mathrm{EP}}_{k}$$

Where ${\text{WAASB}}_{i}$ is the weighted average of absolute scores of the *i*th genotype; $|{\text{IPCA}}_{\mathrm{ik}}$ is the score of the *i*th genotype in the *k*th Interaction Principal Component Axis (IPCA); and ${\mathrm{EP}}_{k}i$ s the explained variance of the *k*th IPCA for *k =* 1,2, …*p*, considering $p=\min\;\left(g-1;e-1\right)$. Genotype and genotype‐environment interaction are considered as random, and Environments and blocks nested within environments are assumed to be fixed factors. This enables the estimation of repeatability (broad‐sense heritability). The BLUPs generated from the WAASB were visualized to explore the genotype‐environment combinations. All the analyses, except otherwise specified, were carried out using the package *metan* (Olivoto and Lúcio 2020) in R 4.4.0 (R Core Team 2024).

#### 2.4.4 GGE Biplot and Multiple‐Trait Index

For each trait, the biplots of the which‐won‐where pattern, mean stability, and environment ranking were generated based on the environment‐centered (G + GE) method with no scaling, and the singular value partitioning was entirely partitioned into the environment eigenvectors. Mega‐environments were identified when all environments fell within the same sector; i.e., mega‐environments were distinguished by having different genotype winners (Yan et al. 2007).

We used the Multi‐trait selection index based on factor analysis and ideotype design (Rocha et al. 2018) to identify stable genotypes across environments and the three quantitative traits, namely fresh biomass yield, leaf length, and leaf width. The analyses were performed using the package *metan* (Olivoto and Lúcio 2020) in R 4.4.0 (R Core Team 2024).

#### 2.4.5 Participatory Variety Selection

Participatory variety selection was implemented to identify farmer‐preferred traits and genotypes. Forty‐one farmers from eight villages participated in the selection exercise on 9 December 2023 in Malanville, when the plants were at the vegetative to flowering stages. On average, five farmers per village were selected based on their willingness, experience in amaranth production and commercialization, and availability to participate in the activity. The farmers were asked to select the genotypes they preferred in their village and provide the reasons for their choices, which provided information about their preferred traits. The number of times a specific genotype and trait were mentioned across villages was used to rank the traits and the genotypes. We assessed the degree of concordance between the ranking by farmers and the multiple trait selection index ranking using Kendall rank correlation in the R *Kendall* package (McLeod 2022).

## 3 Results

### 3.1 Amaranth Genotypes Flowering Time

The genotypes exhibited a diversity of flowering times, ranging from extra‐early genotypes to late flowering. Madiira1 and Madiira2 were late‐flowering, while Nguruma and Poli were extra‐early‐flowering (Table S1). We observed that non‐harvested and harvested (after the first cutting) plants had similar flowering patterns. For instance, early‐flowering genotypes also flowered early during their regrowth after the first cutting.

### 3.2 Within‐Environment Analysis of Variance

**FIGURE 1:**
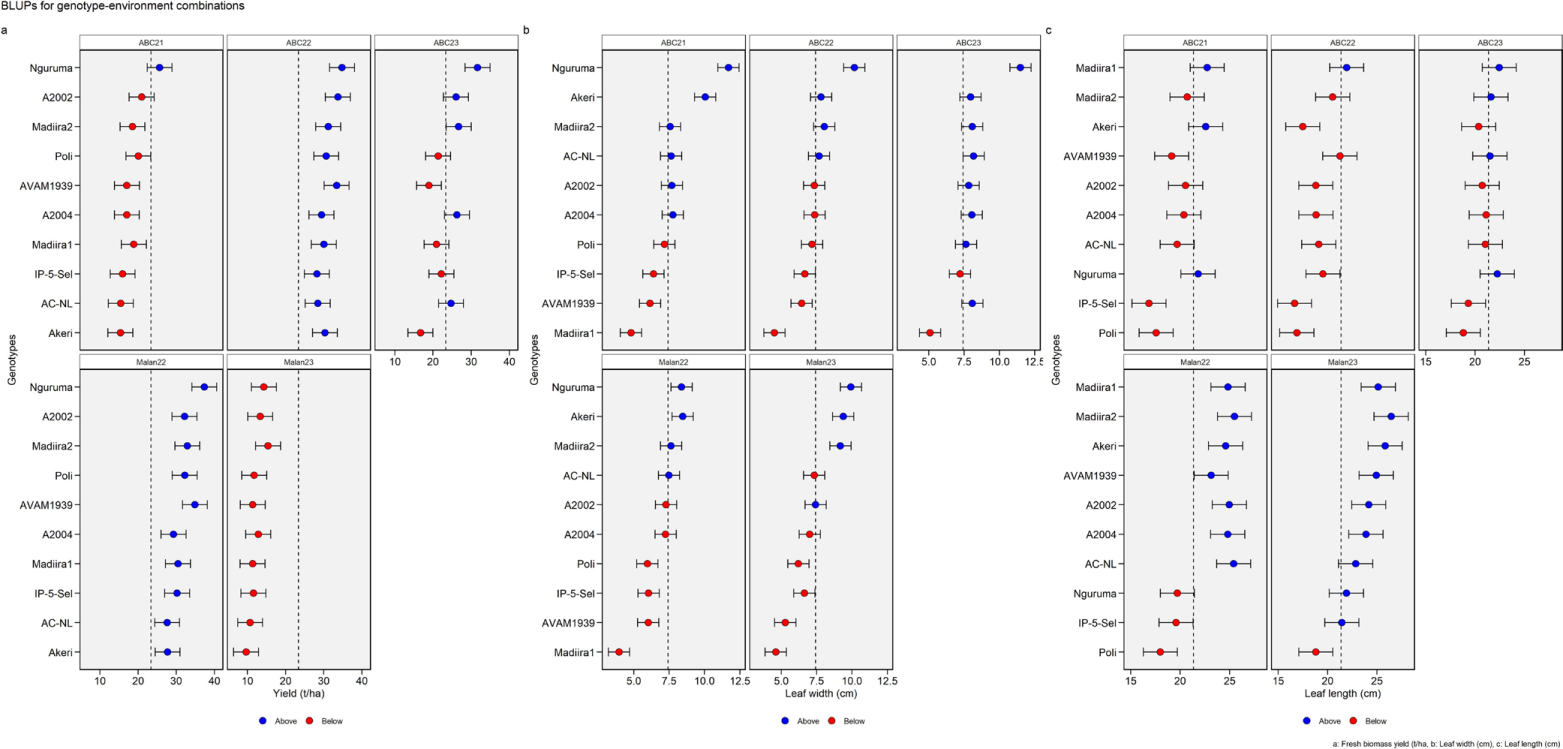
Weighted average scores based on BLUP matrix (WAASB) of (a) fresh biomass yield, (b) leaf width, and (c) leaf length of 10 amaranth genotypes evaluated over five environments in Benin. ABC21, Abomey‐Calavi 2021; ABC22, Abomey‐Calavi 2022; ABC23, Abomey‐Calavi 2023; Malan22, Malanville 2022; Malan23, Malanville 2023.

The highest biomass yields were recorded in 2022 in both Abomey‐Calavi (31.5 t/ha) and Malanville (31 t/ha), while the lowest yield was recorded in Malanville in 2023 (12.3 t/ha) (Figure 1 and Table S2). Nguruma, Madiira2, and A2002 were consistently among the highest‐yielding genotypes across environments (Figure 1).

On average, for all genotypes, the highest leaf width was recorded in ABC21 and ABC23, while the highest leaf length values were recorded in Malan22 and Malan23 (Figure 1 and Table S2). In ABC22 and Malan22, the fresh biomass yield of all genotypes was above average value across the five environments (Figure 1). Madiira1 had the narrowest leaf width, while Nguruma, Akeri, and Madiira2 had the widest leaf width (Figure 1). Overall, Madiira1 and Madiira2 had the longest leaves (Figure 1).

### 3.3 Analysis of Variance and AMMI

The analysis of variance revealed significant differences between genotypes (*p* < 0.001), and the interactions between genotype and environment were also significant (*p* < 0.001) for fresh biomass yield, leaf width, and leaf length. Genotypes' sum of squares accounted for 8.91%, 28.5%, and 73.08% of the total treatment (G + E + G × E) sum of squares for fresh biomass yield, leaf length, and leaf width, respectively (Table 3). Regarding yield, the environment accounted for the highest proportion of the differences, while for leaf width, the difference was most explained by the genotype (Table 3). The broad sense heritability varied from low (0.17) for fresh biomass yield to high for leaf width (0.74). The AMMI model diagnostic suggested AMMI 1, AMMI2, and AMMI2 for fresh biomass yield, leaf length, and leaf width, respectively (Table 3).

**TABLE 3:** AMMI of leaf length, leaf width, and fresh biomass yield.

Source	df	Yield			Leaf length			Leaf width		
		SS	MS	Proportion treatment SS (%)	SS	MS	Proportion treatment SS (%)	SS	MS	Proportion treatment SS (%)
GEN	9	1158.04	128.67***	8.91 (+)	447.85	49.76***	28.5 (+)	316.90	35.21***	73.08 (+)
ENV	4	8148.45	2037.11***	62.71 (+)	428.29	107.07***	27.3 (+)	21.53	5.38***	4.96 (+)
Blocks/Env	10	308.39	30.84 ns	—	40.27	4.03 ns	—	6.65	0.66*	—
GxE	36	1554.36	43.18**	11.96 (+)	381.56	10.60***	24.31 (+)	62.41	1.73***	14.39 (+)
IPC1	12	1028.13	85.68**	66.15 (−)	232.82	19.40***	61.02 (−)	33.01	2.75***	52.90 (−)
IPC2	10	298.95	29.90 ns	19.23 (−)	90.26	9.03**	23.66 (−)	20.93	2.09***	33.54 (−)
IPC3	8	146.06	18.26 ns	9.40 (−)	45.47	5.68	11.92 (−)	7.35	0.92*	11.77 (−)
IPC4	6	81.22	13.54 ns	5.22 (−)	13.01	2.17 ns	3.41 (−)	1.12	0.19	1.80 (−)
Residuals	90	1823.74	20.26		271.79	3.02		26.14	0.29	
Broad sense heritability		0.17			0.32			0.74		

*Note:* *, **, *** means significant at 0.05, 0.01 and 0.001, respectively, ns = not significant (*p* > 0.05); +: Proportion of the total treatment (G + E + GxE) sum of square explained by each source of variation. −: Proportion of the sum of the total treatment (GxE) square of GxE explained by each IPC.

Abbreviations: ENV, environment; GEN, genotypes; IPC, interactive principal component; REP, replication.

### 3.4 GGE Biplot of Amaranth Fresh Biomass Yield, Leaf Length and Leaf Width

#### 3.4.1 Yield

**FIGURE 2:**
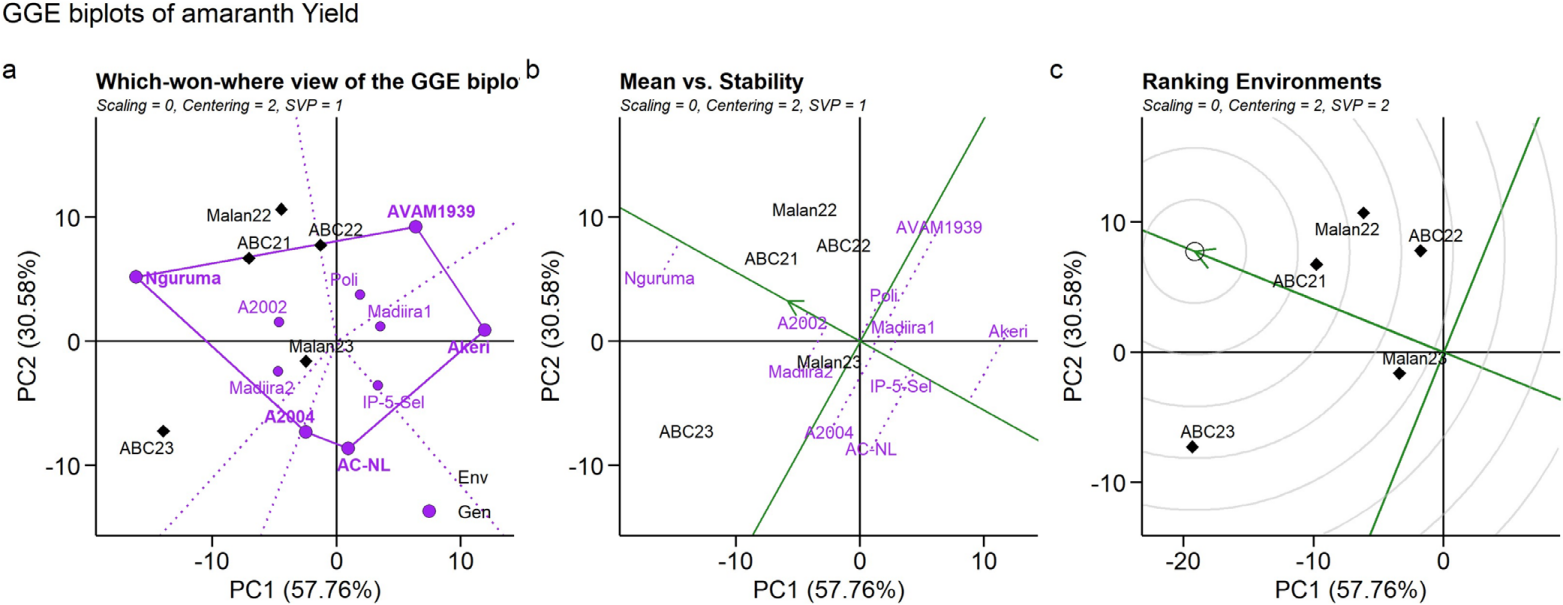
Environment‐by‐Genotype interactions: (a) Which‐won‐where, (b) Mean Vs stability, and (c) Ranking environments of fresh biomass yield in 10 amaranth genotypes evaluated across five environments in Benin. ABC21, Abomey‐Calavi 2021; ABC22, Abomey‐Calavi 2022; ABC23, Abomey‐Calavi 2023; Malan22, Malanville 2022; Malan23, Malanville 2023.

The first two principal components explained 88.34% of the genotype and G × E interaction variation of fresh biomass yield, suggesting that the biplot based on PC1 and PC2 indicates a good goodness of fit. All environments fell within the same sector, suggesting that, to a rank‐two approximation, the same genotype, i.e., Nguruma, performed best in all environments (Figure 2a).

An ideal genotype should combine high performance and high stability for the trait of interest. The arrow on the axis of the Average Environment Coordination (AEC) abscissa indicates the direction of higher mean performance for the genotypes (Figure 2b). Genotypes with the lowest projection onto the AEC ordinate are the most stable, which indicates their highly consistent rank across the five environments. A2002, IP‐5‐Sel, Madiira1, and Nguruma were the most stable for fresh biomass yield, while AVAM1939 and A2004 were the least stable genotypes for the same trait (Figure 2b). ABC21 was the most representative of the test environments and showed strong discriminativeness among the genotypes. It can be considered an ideal environment for selecting superior genotypes. Malan23 had a short vector and was less discriminating as it provided little information about the genotypes. ABC23 had long vectors but a large angle with the AEC and could not be used to select superior genotypes, but to cull unstable genotypes. ABC23 was more discriminative than ABC22 and Malan22, which had moderate vector length and large angles with the AEC (Figure 2c). This environment can be used to cull unstable genotypes.

#### 3.4.2 Leaf Width

**FIGURE 3:**
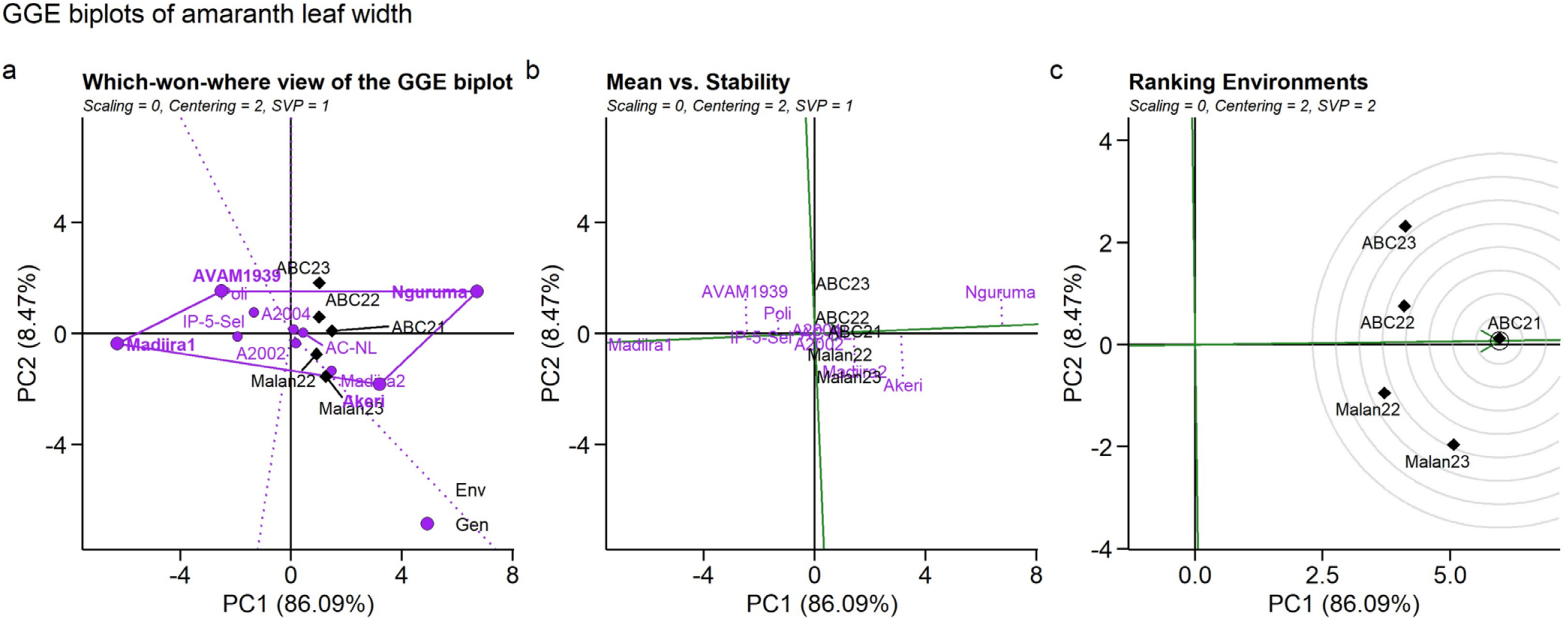
Environment‐by‐Genotype interactions: (a) Which‐won‐where, (b) Mean Vs stability, and (c) Ranking environments of leaf width in 10 amaranth genotypes evaluated across five environments in Benin. ABC21, Abomey‐Calavi 2021; ABC22, Abomey‐Calavi 2022; ABC23, Abomey‐Calavi 2023; Malan22, Malanville 2022; Malan23, Malanville 2023.

The first two principal components accounted for 94.56% of the variation in leaf width due to genotype and genotype × environment interaction. This suggests that the biplot based on PC1 and PC2 provides a good representation of the data set, indicating a good goodness of fit. Like the fresh biomass yield, all environments fell within the same sector for leaf width, with Nguruma as the winning genotype (Figure 3a).

IP‐5‐Sel, A2004, AC‐NL, Madiira1, and Poli were the most stable genotypes regarding leaf width. On the contrary, AVAM1939, Madiira2, and Nguruma were less stable (Figure 3b). All the environments provided information on the genotypes as they had long vectors. However, ABC21 was the most discriminative and representative environment for leaf width (Figure 3c). Nguruma had the widest leaf, followed by Akeri and Madiira2 (Figure 3c).

#### 3.4.3 Leaf Length

**FIGURE 4:**
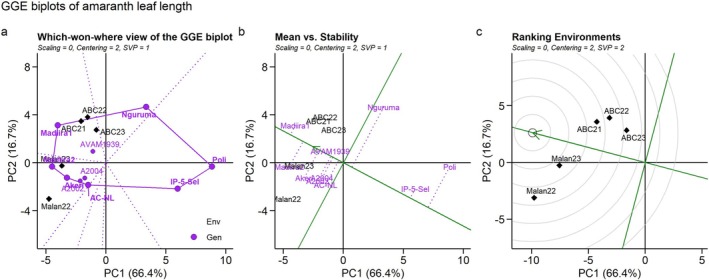
Environment‐by‐Genotype interactions: (a) Which‐won‐where, (b) Mean Vs stability, and (c) Ranking environments of leaf length in 10 amaranth genotypes evaluated across five environments in Benin. ABC21, Abomey‐Calavi 2021; ABC22, Abomey‐Calavi 2022; ABC23, Abomey‐Calavi 2023; Malan22, Malanville 2022; Malan23, Malanville 2023.

The first two principal components explained 83.1% of the genotype and genotype × environment interaction variation of leaf length, suggesting that the biplot based on PC1 and PC2 provides a good representation of the data set and indicates a good goodness of fit. ABC21, ABC22, and ABC23 fell within a single vector with Madiira1 as the winning genotype, while Malan22 and Malan23 fell within the same sector with Madiira2 as the winning genotype (Figure 4a).

IP‐5‐Sel and Madiira1 were the most stable genotypes, while Nguruma and Poli were the least stable (Figure 4b). Madiira1 had the longest leaf, followed by Madiira2, Akeri, and AVAM1939 (Figure 4b). Malan22 and Malan23 were the most representative and discriminative environments for leaf length, respectively (Figure 4c). Malan23 combined strong representativeness of test environments and discrimination of the genotypes, and could be an ideal environment for amaranth leaf length evaluation.

### 3.5 Multi‐Trait Selection Index

**FIGURE 5:**
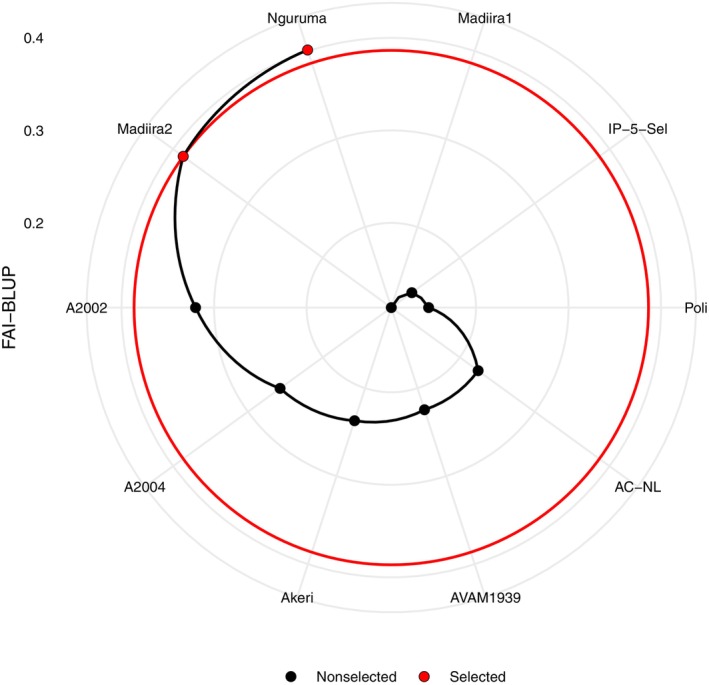
Classification of amaranth genotypes evaluated across five environments in Benin using multi‐trait selection (FAI‐BLUP) index.

The multi‐trait selection index ranks genotypes based on their performance across multiple traits and environments. Our study examined three key traits: fresh biomass yield, leaf width, and leaf length. Based on these three traits, the multi‐trait selection index ranks the genotypes as follows: Nguruma>Madiira2>A2002>A2004>Akeri>AC‐NL, AVAM1939>Poli>IP‐5‐Sel>Madiira1 (Figure 5). Nguruma and Madiira2 were identified as the most stable genotypes considering the traits of interest (Figure 5).

### 3.6 Participatory Selection

The most preferred traits by farmers included ramification (high number of branches), market acceptability, late flowering, and cooking ability. Other essential traits driving farmers' varietal preferences included leaf size, green to dark green leaf color, seed production, leaf yield, and color retention after cooking. However, early flowering and narrow leaves were not preferred (Table 4). Based on the preferred traits, farmers selected Madiira2, AC‐NL, Akeri, and Nguruma as the top‐performing varieties (Table S3). It is worth noting that farmers also listed cooking‐ and palatability‐related traits based on their perception rather than what could actually be measured and directly observed in the field. Comparing the multiple trait and farmer rankings with Kendall's tau statistic gave a 0.47 (*p* = 0.082), indicating a moderate association between both rankings.

**TABLE 4:** Preferred amaranth traits listed by farmers.

Traits	Rank of the traits
Ramifications (high number of branches)	1
Market acceptability^a^	1
Late flowering	2
Good cooking quality (less softer after cooking)	3
High leaf yield	3
High seed yield	3
Retention of leave color after cooking	4

^a^ From discussions with participants, marketability is market acceptability based on leaf characteristics (color, size, shape).

## 4 Discussion

### 4.1 Identification of Adapted and Stable Genotypes

The differences in performance among genotypes for the traits of interest within the same environment suggest that there genetic variability among the genotypes. This offers the opportunity to identify genotypes adapted to various growing conditions and meet the preferences of different stakeholders along amaranth value chains. Madiira1 was the genotype with the longest and narrowest leaf, which aligns with a previous report in Kenya and Tanzania (Dinssa et al. 2022). Madiira1 and Madiira2 were late‐flowering, consistent with previous findings (Dinssa et al. 2022; Dinssa et al. 2019). Late flowering is essential trait for vegetable amaranth growers who practice repeated harvesting (cuttings) from the same plants because it extends the number of harvests, thereby increasing the biomass yield per plant. Early flowering reduces new leaves' appearance and quality (Grubben 2004). However, late flowering is a less desirable trait in seed production as this prolongs the production cycle and increases production costs. Besides, late‐flowering genotypes like Madiira1 and Madiira2 have low seed yield, which is a constraint for seed producers as this would increase seed production costs and, subsequently, seed prices (Dinssa et al. 2018, 2022).

The AMMI revealed significant genotype‐by‐environment interactions for fresh biomass yield, leaf length, and leaf width. The BLUP‐based method (WAASB) output supports this finding and helped reveal the crossover nature of the interactions (based on the first component). The BLUP‐based method and GGE biplots provided similar information on the adaptability and performance of the genotypes. The five environments shared the same winning genotype for fresh biomass yield and leaf width and can be grouped into one mega‐environment (Sserumaga et al. 2018; Abakemal et al. 2016). Previous reports on genotype‐by‐environment interactions for vegetable amaranths in Tanzania identified two mega‐environments characterized by hot and cool weather (Dinssa et al. 2019). However, despite differences in environmental conditions (temperatures, soil types, rainfall), the five environments did not cluster into different mega‐environments. This finding suggests the environmental variations are not high enough to induce considerable changes in the genotypes' responses. The identification of a mega‐environment implies that further testing can be done in fewer environments. We identified the least stable genotypes for leaf width (AVAM1939, Madiira2, and Nguruma), leaf length (Nguruma and Poli), and fresh biomass yield (AVAM1939 and A2004), suggesting that these genotypes exhibit higher sensitivity to unpredictable environmental factors like variations in rainfall, temperature, and soil conditions This implies that reliable recommendations for these genotypes in cultivation may require additional testing environments. The environment ABC21, characterized by a ferralitic soil, average minimum and maximum temperatures of 25°C and 28°C, respectively, photosynthetically active radiation between 70 and 110, and relative humidity between 85% and 90%, was the most suitable for selecting superior amaranth genotypes for yield and leaf width. Evaluating amaranth genotypes in environments with similar conditions to ABC21 will help select superior genotypes. However, the relatively low and moderate repeatability recorded for fresh biomass yield and leaf length suggests that reliable selection for these traits will require more testing environments. Beyond environmental factors, management practices such as soil fertility and water management can affect the performance of genotypes and require further investigation for the recommendation of a technology package consisting of agronomy practices and varieties.

### 4.2 Moderate Association Between Farmers' Ranking and Multi‐Trait Selection

We identified farmer‐preferred traits in amaranth. These traits included fresh biomass yield, flowering time, leaf traits (leaf size, color, color retention after cooking, texture after cooking), and marketability. Beyond productivity traits, marketability and consumer preference traits were identified as important traits by farmers (Dinssa et al. 2018). This revealed that farmers not only select varieties to maximize productivity but also to align their choices with what consumers and buyers are willing to pay for (Wanyama et al. 2023). Most villages selected Madiira2 as the best genotype due to its marketability, late flowering, high ramification, market acceptability, perceived cooking ability, and appealing leaves (light green color, moderately large, and long leaves). Voss et al. (2025) identified partly different preferred amaranth traits such as plant survival, leaf size, yield, taste, and marketability as the main traits driving the varietal choice of farmers in Benin, Mali, and Tanzania, with A2004 identified as the most accepted genotyoe across the various market segments, followed by AC‐NL and Poli. The study by Voss et al. (2025) provided overall preferences across Benin, Mali, and Tanzania, but did not show traits preference within each country.

Farmers associated ramification with the regeneration ability linked to the number of harvests and biomass production. In Tanzania and Kenya, farmers also listed multiple harvests and broad leaves as important amaranth traits (Dinssa et al. 2022). However, beyond this productivity trait, they factored in all the listed traits to select their preferred genotypes. For instance, Nguruma was identified as the most productive genotype based on farmer assessment and agronomic evaluation data. Nguruma is one of the most popular amaranth varieties in Tanzania, as farmers have reported it to be drought‐tolerant (Wanyama et al. 2023). However, Nguruma was not ranked as the most preferred genotype because of its dark green color and growth habit, which some farmers consider “weed.” This finding is similar to that of Voss et al. (2025), who found that Nguruma was part of the less accepted genotypes across the gender‐based market segments, despite being reported as very productive. The preferred genotypes combined high productivity with leaf quality traits.

Madiira1 was identified as the least performing genotype considering both the agronomic traits and the farmers' ranking. Similarly, Voss et al. (2025) identified Madiira1 as the least accepted amaranth variety across gender‐based market segments in Benin, Mali, and southern Tanzania. However, Madiira1, although not exceptionally productive, is highly preferred in at least eastern and northern Tanzania (Dinssa et al. 2020, 2022), while it was the least preferred genotype in Benin. However, Poli, a less preferred genotype in our study, was identified as one of the most preferred genotypes, especially by young and older women across Benin, Mali, and Tanzania (Voss et al. 2025). Poli is an extra‐early variety and does not fit locations that practice repeated harvesting of the same plant, but it is well‐suited to uprooting production systems due to its early growth vigor getting ready for uproot harvesting about 30 days after sowing, a practice widely used in urban and per‐urban areas in Tanzania and Kenya. A2002 and A2004 have been released in Mali, and their seed can be produced and marketed in Benin (ECOWAS 2008). Except for Madiira1, which has narrow leaves, and Poli, which is extra early in flowering and is less preferred by farmers, we recommended Nguruma, Madiira2, Akeri, AC‐NL, AVAM1939, and IP‐5‐Sel for release in Benin based on this study.

The relatively low number of farmers involved in the participatory selection may limit the generalization of their variety preferences to the amaranth‐growing areas in Benin. The participants were, however, selected based on their experience in amaranth production and commercialization, so they know producers and market needs. Besides, the farmers were from different villages to ensure the representativeness of preferences in the region. Additionally, as the experiments were conducted in specific locations, this could limit the scope of inference to other amaranth‐growing areas in Benin. However, these two locations are situated in contrasting climatic regions: the Guinean (Abomey‐Calavi) and the Sudanian (Malanville) (Adomou 2005), representing the major amaranth‐growing areas in the country. As such, the study's findings remain valid for recommending amaranth varieties in major growing areas of Benin.

## 5 Conclusion

Considering their agronomic performance, we identified Nguruma and Madiira2 as the top performers across environments and years in Benin. These genotypes also demonstrated good stability in terms of fresh biomass yield. Farmers preferred genotypes with many branches, market‐accepted leaves, and late flowering. Thus, besides Nguruma and Madiira2, we identified additional genotypes, including AC‐NL, Akeri, AVAM1939, and IP‐5‐Sel, as the top‐performers based on farmers' preferences. The findings of this study guided the selection of amaranth genotypes that have not yet been released in the ECOWAS region for release and registration in Benin.

## Conflicts of Interest

The authors declare no conflicts of interest.

## Supporting information


**Table S1:** Flowering of amaranth genotypes in a multilocation trial (five environments) in Benin.
**Table S2:** Individual analysis of variance, environment mean of biomass yield, leaf length, and leaf width of 10 amaranth genotypes across environments.
**Table S3:** Ranking of 10 amaranth genotypes by farmers*.

**Data S1:** Dataset of the evaluation of 10 amaranth genotypes in two locations over three seasons in Republic of Benin.

## Data Availability

The data that supports the findings of this study are available in the Supporting Information—S1 of this article.
