# Supplementary material for: Agronomic Performance and Stability of Vegetable Amaranth (Amaranthus spp.) Genotypes in Benin
Source: Plant Environ Interact. 2025 Aug 7;6(4):e70076. doi: 10.1002/pei3.70076 (PMC12329764; doi:10.1002/pei3.70076)
Supplement: Supplementary file 1 — Table S1: Flowering of amaranth genotypes in a multilocation trial (five environments) in Benin. Table S2: Individual analysis of variance, environment mean of biomass yield, leaf length, and leaf width of 10 amaranth genotypes across environments. Table S3: Ranking of 10 amaranth genotypes by farmers*. [file PEI3-6-e70076-s002.docx]

**Supplementary Tables**

**Table S1:** Flowering of amaranth genotypes in a multilocation trial (five environments) in Benin

| **Genotypes** | **Flowering** |
| --- | --- |
| Madiira1 | Late |
| Madiira2 | Late |
| Akeri | Medium |
| Nguruma | Extra early |
| Poli | Extra early |
| IP-5-Sel | Early |
| AVAM1939 | Medium |
| AC-NL | Early |
| A2002 | Early |
| A2004 | Early |
| Local | Medium |

**Table S2:** Individual analysis of variance, environment mean of biomass yield, leaf length, and leaf width of 10 amaranth genotypes across environments

| Environments | Fresh biomass yield | | | Leaf length | | | Leaf width | | |
| --- | --- | --- | --- | --- | --- | --- | --- | --- | --- |
|  | Mean±Se | Mean Square | CV (%) | Mean±Se | Mean Square | CV (%) | Mean±Se | Mean Square | CV (%) |
| ABC21 | 17.96±0.95 | 57.85* | 22.23 | 19.98±0.46 | 16.007** | 8.97 | 7.59±0.34 | 12.778*** | 7.31 |
| ABC22 | 31.01±0.95 | 28.917ns | 16.59 | 19.09±0.43 | 12.841*** | 7.30 | 7.32±0.26 | 6.050*** | 5.97 |
| ABC23 | 24.02±1.27 | 142.967*** | 16.07 | 20.91±0.31 | 4.348ns | 7.41 | 7.92±0.26 | 7.583*** | 4.95 |
| Malan22 | 31.48±1.17 | 58.771ns | 18.71 | 23.06±0.65 | 36.477*** | 5.35 | 6.85±0.26 | 5.613*** | 7.34 |
| Malan23 | 12.3±0.73 | 12.874ns | 24.42 | 23.47±0.62 | 22.479** | 10.36 | 7.38±0.34 | 10.121*** | 10.03 |

Se : standard error; CV: Coefficient of variation, ; *,**,*** means significant at 0.05, 0.01 and 0.001, respectively”, ns = not significant (P>0.05).

**Table S3:** Ranking of 10 amaranth genotypes by farmers*

| Genotypes | Rank | Localities | Reasons for variety selection |
| --- | --- | --- | --- |
| Madiira2 | 1 | Madecali, Garou Zenon, Bodjecali, Malanville, Kotchi, Banite | Late flowering, high number of branches, less soft after cooking, High marketability |
| AC-NL | 2 | Monkassa, Bodjecali, Kotchi, Banite | High marketability, high number of branches, late flowering, large leaves |
| Akeri | 3 | Madecali, Malanville, Toumboutou | Green leaf color, high number of branches, plant height, retention of leave color after cooking |
| Nguruma | 3 | Bodejcali, Banite, Kotchi | High leaf yield, less soft after cooking, high marketability |
| IP-5-Sel | 5 | Monkassa, Banite | High number of branches, high seed production |
| A2002 | 5 | Garou Zenon, Malanville | High marketability, high number of branches, late flowering, high seed production |
| A2004 | 7 | Toumboutou | High marketability |
| AVAM1939 | 8 | Not selected in any villages | Early flowering |
| Poli | 8 | Not selected by farmers from any village | Early flowering |
| Madiira1 | 8 | Not selected by farmers from any village | Narrow leaves |

* Ranking and trait preferences recorded from each village
